# Norepinephrine as a Potential Aggravator of Symptomatic Cerebral Vasospasm: Two Cases and Argument for Milrinone Therapy

**DOI:** 10.1155/2014/630970

**Published:** 2014-11-09

**Authors:** F. A. Zeiler, J. Silvaggio, A. M. Kaufmann, L. M. Gillman, M. West

**Affiliations:** ^1^Section of Neurosurgery, University of Manitoba, GB-1 820 Sherbrook Street, Winnipeg, MB, Canada R3A 1R9; ^2^Section of Critical Care, Department of Medicine, University of Manitoba, Room GC425, 820 Sherbrook Street, Winnipeg, MB, Canada R3T 2N2

## Abstract

*Background.* During hypertensive therapy for post-subarachnoid hemorrhage (SAH) symptomatic vasospasm, norepinephrine is commonly used to reach target blood pressures. Concerns over aggravation of vasospasm with norepinephrine exist. *Objective.* To describe norepinephrine temporally related deterioration in neurological examination of two post-SAH patients in vasospasm. *Methods.* We retrospectively reviewed two charts of patients with delayed cerebral ischemia (DCI) post-SAH who deteriorated with norepinephrine infusions. *Results.* We identified two patients with DCI post-SAH who deteriorated during hypertensive therapy with norepinephrine. The first, a 43-year-old male presented to hospital with DCI, failed MABP directed therapy with rapid deterioration in exam with high dose norepinephrine and MABP of 140–150 mm Hg. His exam improved on continuous milrinone and discontinuation of norepinephrine. The second, a 39-year-old female who developed DCI on postbleed day 8 responded to milrinone therapy upfront. During further deterioration and after angioplasty, norepinephrine was utilized to drive MABP to 130–140 mm Hg. Progressive deterioration in examination occurred after angioplasty as norepinephrine doses escalated. After discontinuation of norepinephrine and continuation of milrinone, function dramatically returned but not to baseline. *Conclusions.* The potential exists for worsening of DCI post-SAH with hypertensive therapy directed by norepinephrine. A potential role exists for vasodilation and inotropic directed therapy with milrinone in the setting of DCI post-SAH.

## 1. Introduction

Delayed cerebral ischemia (DCI) post-subarachnoid hemorrhage (SAH) poses significant challenges to the treating physician. Current standard therapy consists of hypertensive directed measures in order to ensure preservation of cerebral blood flow to risk territories and is titrated typically to the neurological examination [1–3]. In addition to this, maintenance of euvolemia is also a cornerstone of therapy [2]. In order to achieve the desired level of hypertension, a variety of inotropic and vasoconstrictive vasopressors have been utilized in the literature.

Despite our best attempts at preservation of cerebral blood flow with hypertensive therapy, some patients progress to develop cerebral infarcts with a range of morbidity and mortality depending on infarct location and extent [3].

Despite maximal therapy, some patients will fail hypertensive direct therapy for DCI post-SAH. However, with the use of peripheral vasoconstrictive substances to achieve desired mean arterial blood pressure (MABP) goals, one could question their effect on aggravating the cerebral vasoconstriction and having a negative impact on the neurological exam. To date, animal literature points towards cerebral vasoconstriction with the administration of norepinephrine [4–6]. Autopsy evidence suggests a dose dependant vasoconstrictive effect on proximal cerebral vessels with norepinephrine administration [7]. Furthermore, norepinephrine levels are known to be elevated in the cerebral spinal fluid of SAH patients and have been postulated as a potential mediator of cerebral vasospasm post-SAH [8]. Similarly, healthy human controls have displayed cerebral vasospasm via transcranial Dopplers (TCDs) when given intravenous norepinephrine [9]. Thus, norepinephrine may not be a safe choice for MABP directed therapy in post-SAH symptomatic cerebral vasospasm.

Within this paper we describe two cases of DCI post-SAH with worsening neurological examination temporally related to elevation of norepinephrine doses during hypertensive therapy. These effects were ameliorated via discontinuation of norepinephrine and continuing intravenous phosphodiesterase inhibitor (PDEI) based therapy with milrinone.

## 2. Case Presentations

### 2.1. Case 1

A 43-year-old male with no significant past medical history presents to hospital with a 4-day history of headache, lethargy, and diplopia. Computed tomography (CT) of the brain demonstrated a Fisher grade 4 SAH; clinically he was Hunt and Hess grade 2 SAH. CT-angiography displayed a left posterior communicating artery aneurysm and left middle cerebral artery (MCA) vasospasm.

Clinically he had a partial oculomotor nerve palsy on the left and a significant headache. He was admitted, with plans for clipping of the aneurysm in the morning. Nimodipine prophylaxis was started. On postbleed day 5, he was seen on morning rounds neurologically unchanged, awaiting surgery. Within 30 minutes he developed progressive dysphasia, both receptive and expressive, with right hemineglect. Repeat CT head failed to display signs of aneurysmal rebleed. He received 2.5 liters of crystalloid and was transferred to the intensive care unit (ICU) for hypertensive therapy prior to going to the operative room (OR). Norepinephrine was started at 0.02 mcg/kg/min and titrated to 0.16 mcg/kg/min to achieve MABP of 130 mm Hg, as measured with the arterial line transducer zeroed at the tragus. Significant deterioration followed this titration of norepinephrine, progressing to right hemiplegia and aphasia.

The patient was then taken to the OR and the aneurysm was clipped successfully. Intraoperatively significant left MCA vasospasm was noted. The vessels were wiped with papaverine soaked gel foam. Mean arterial pressures were maintained between 130 and 140 mm Hg intraoperatively, with no hypotensive episodes noted in the anesthesia records.

Postoperatively in the ICU, the patient awoke with right hemiplegia, right homonymous hemianopsia, and aphasia while achieving the target MABP of 150 mm Hg with norepinephrine at 0.68 mcg/kg/min. It was elected to stop norepinephrine and start intravenous milrinone therapy 45 minutes after emergence from his general anesthetic. A 5 mg bolus of milrinone over 10 minutes was given, followed by a continuous infusion of 0.75 mcg/kg/min. Within 30 minutes of discontinuing the norepinephrine and starting milrinone all deficits, except the third nerve palsy, resolved. His MABP at this time was 95 mm Hg.

On postbleed day 6, after 24 hours of milrinone therapy and a stable neurological examination, he developed acute sensory extinction on the right side and hemianopsia. Milrinone was rebolused, and the infusion was increased to 1.5 mcg/kg/min with partial improvement in symptoms within 40 minutes. Mean arterial pressures at this time were 120 mm Hg. Given only a partial response to milrinone escalation, norepinephrine was restarted with a goal MABP of 140 mm Hg, and interventional neuroradiology was called for angioplasty. While waiting for angioplasty, the norepinephrine was titrated to 0.46 mcg/kg/min with progressive deterioration in the neurological examination leading to hemiplegia.

The angioplasty of a severely spastic left proximal MCA and anterior cerebral artery (ACA) was conducted without incident. The degree of spasm in these arterial segments was noted to be worse compared to previous CT-angiography. Distal small vessel spasm in the left MCA territory was noted as well during angioplasty.

After angioplasty he remained hemiparetic, with hemianopia and dysphasia, while the norepinephrine remained infusing with the goal of achieving MABP of 140 mm Hg. With no improvement in the next 48 hours, the norepinephrine was discontinued, while milrinone was continued at 1.5 mcg/kg/min with MABP around 90 to 110 mm Hg. The patient improved over the following hours after discontinuation of norepinephrine. Milrinone was then titrated down by 0.25 mcg/kg/day increments.

Currently, on postbleed day 25 he only has a partial third cranial nerve palsy and mild expressive language issues. Euvolemia was targeted throughout his entire course. No cardiac output monitoring was conducted throughout the patient's ICU stay.

### 2.2. Case 2

A 39-year-old female with a past medical history significant for polio, leaving her wheelchair bound secondary to lower extremity involvement, was admitted for a history of acute onset headache and mild confusion. She had a CT of the brain demonstrating a Fisher grade 4 SAH; clinically she was Hunt and Hess grade 2 SAH. Her CT-angiography demonstrated a right MCA aneurysm. She was taken to the OR for microsurgical clipping of the aneurysm and external ventricular drain (EVD) placement.

Postoperatively the patient did initially well. No focal deficits were noted postoperatively other than a partial left abducens palsy related to fluctuations in intracranial pressure (ICP).

On postbleed day 7 a follow-up CT-angiogram was performed displaying right MCA and ACA vasospasm, but the patient was clinically unaffected at that time. On postbleed day 8, significant diuresis and cerebral salt wasting were noted. On postbleed day 9 the patient developed left upper motor neuron (UMN) facial weakness and left arm paresis. She was taken to ICU for management of her DCI.

Milrinone therapy was initiated. Five milligrams of milrinone was bolused over 10 minutes, followed by a continuous infusion at 0.75 mcg/kg/min. She improved within 20 minutes to baseline. On postbleed day 11 she deteriorated again in a similar fashion. Milrinone was then rebolused, and the infusion was increased to 1.5 mcg/kg/min, leading to neurological improvement with minor residual pronator drift.

On postbleed day 13, neurological deterioration occurred again with now left UMN facial weakness and left arm paresis. Norepinephrine was started at 0.02 mcg/kg/min to target MABP of 140 mm Hg and interventional radiology was called for angioplasty. The proximal MCA and ACA were noted to be severely spastic and were the targets of angioplasty. These vessels appear as narrowed, compared to preangioplasty CT-angiography. Distal small vessel spasm was unchanged. The angioplasty occurred without incident and a good angiographic result was obtained.

Neurologically after angioplasty the patient's left arm was plegic. Norepinephrine was increased to 0.22 mcg/kg/min to target MABP of 150 mm Hg. Occasional episodes of improvement were seen in the left arm but not sustained. Episodes of symptomatic pulmonary edema began to develop during this phase of her treatment. An MRI was conducted displaying watershed diffusion weighted changes in the ACA/MCA and MCA/posterior cerebral artery (PCA) territories. There was some perfusion/diffusion mismatch noted, and thus aggressive MABP therapy was continued for these potentially salvageable areas. No improvement occurred over the following 72 hours.

Norepinephrine was then discontinued on postbleed day 16, with milrinone therapy running at 1.5 mcg/kg/min. The norepinephrine was discontinued given concerns over potential aggravation of cerebral vasoconstriction and the presence of maintained MABP goals on milrinone alone. No cardiac output monitoring was conducted during the patient's ICU stay. Within 12 hours of discontinuing norepinephrine, the patients left arm went from plegic to 4/5 strength in biceps, with 2-3/5 strength throughout the other muscles. Her left UMN facial weakness almost completely resolved during this time frame. Milrinone was then titrated off over the following day, as described in the first case.

Currently, on postbleed day 22 the patient has the same examination with mild left UMN facial weakness and the paretic left arm.

## 3. Discussion

Cerebral vessels are known to be innervated by nerves containing norepinephrine and 5-hydroxytryptamine (5-HT) [4]. Norepinephrine has been demonstrated in both animal and human autopsy specimens, to induce a dose mediated vasoconstrictive effect on cerebral vasculature [7].

Norepinephrine mediates vasoconstriction of cerebral vessels via two mechanisms. First, at low serum concentrations the alpha adrenergic mediated effects of norepinephrine lead to vasoconstriction. Second, at high concentrations, low-affinity sites [7] or gamma adrenoreceptor [8, 9] may be stimulated by norepinephrine leading to a much more pronounced cerebral vasoconstriction. These effects are seen in the proximal large cerebral vessels greater than in the smaller distal vessels [7].

In the setting of aneurysmal SAH, both serum and CSF norepinephrine levels have been demonstrated to be elevated [10, 11]. These serum [11] and CSF levels [10] seem to rise correlating with the severity of SAH and may be related to symptomatic cerebral vasospasm or just a marker of disease severity.

During hypertensive therapy for DCI post-SAH, a variety of vasopressors have been utilized, with norepinephrine displaying the most predictable hemodynamic response in traumatic brain injury as identified in recent literature [12–14]. However there are concerns of cerebral vasoconstriction with norepinephrine administration, as outlined above. Within the two cases described, we were able to temporally relate worsening of the neurological examination with escalating norepinephrine doses during the treatment of their DCI. Though no objective radiographic markers of worsening cerebral vasospasm were identified (other than during angioplasty in Case 1), the deterioration of the neurological examination was dramatic and partially/entirely reversible with discontinuation of the norepinephrine infusions. With the absence of cardiac output monitoring and objective measures of cerebral blood flow, we can only suggest the temporally related decompensation was potentially related to norepinephrine induced vasoconstriction. Furthermore, the potential utility of the PDEI milrinone via continuous intravenous infusions was displayed in both of these cases.

A few important points can be taken from both of these cases. First, MABP directed therapy for DCI postaneurysmal SAH can be challenging. It is not uncommon, as seen in both cases, that there exists a balancing act between symptomatic improvement with pharmacological alterations in cerebral blood flow and inducing systemic hemodynamic complications, such as pulmonary edema. Second, there seemed to be a norepinephrine dose related temporal change in both patients' neurological examinations. One patient displayed this deterioration with escalating norepinephrine both on and off milrinone therapy. The second patient displayed this effect while being on milrinone. Caution should be taken however, since the deteriorations seen may reflect either failure of hypertensive therapy (i.e., refractory DCI) or hypertension induced posterior reversible encephalopathy syndrome (PRES) related to escalating norepinephrine doses. Given the temporal relation between deterioration/improvement and norepinephrine doses, with neuroimaging failing to demonstrate features of PRES, we feel medication induced vasoconstriction is a real possibility. Third, this potential norepinephrine mediated deterioration is seemingly reversible to some extent, as seen when both patients had improvement in their neurological examination with discontinuation of norepinephrine. However, this improvement is clouded by the fact that both were on high dose intravenous infusions of milrinone at the time. Finally, the role of vasodilator therapy utilizing PDEI, such as milrinone, via continuous intravenous infusions is still undefined, though, in the two cases described and in existing literature [15], continuous infusions of milrinone have been shown to lead to neurological improvement in DCI post-SAH. The concerns over aggravation of cerebral vasospasm do not exist with milrinone, as they do with norepinephrine. Thus, maybe vasodilator therapies should be the front line for DCI post-SAH. Further study in this area is warranted.

There are significant limitations to our study. First, we only have two cases and this hardly is proof of concept that norepinephrine leads to worsening of DCI in SAH. This is further exemplified by the numerous other cases treated at both our institution and others, utilizing MABP directed therapy with norepinephrine, which have failed to display this response. This raises the point of a potential genetic driver of cerebrovascular response to particular vasopressors. Genetic polymorphisms within adrenoreceptors have been increasingly linked to hypertension and cardiovascular disease [16]. For example, the alpha 2B adrenergic insertion/deletion polymorphism has demonstrated greater degrees of peripheral vasoconstriction when stimulated with an alpha 2 agonist, dexmedetomidine, compared to controls [17]. Furthermore, this polymorphism has been linked to sudden cardiac death, presumably related to coronary vasospasm [18]. Similarly, alterations in the beta 2 adrenoreceptor have been linked to refractory hypertension, related to impaired vasodilatory effects when stimulated. Endothelin-1, angiotensin II, and nitric oxide-cGMP pathway polymorphisms have also been linked to altered adrenergic vascular reactivity and are associated with hypertension [16]. All of the mentioned polymorphisms could contribute to undesired cerebrovascular reactivity to adrenergic stimulation with vasopressor agents. However, insufficient data exists in the literature to date to make further comments on this. Second, the only data that we have to implicate norepinephrine in worsening cerebral vasospasm is the direct temporal relationship between administration of this drug and deterioration of the neurological examination. Only during angioplasty in Case 1 was the potential for worsening of proximal vasospasm, compared to CT-angiography, made. Though we suspect that this deterioration is related to worsening vasospasm, given the outlined mechanisms of action of norepinephrine on cerebral vasculature, we have no objective imaging evidence of this. The deterioration in these two cases could very well be a demonstration of failure of norepinephrine therapy in DCI or PRES (as previously mentioned), as opposed to worsening vasoconstriction related to norepinephrine. Potentially functional MRI or even Xenon enhanced CT would have shed more light on the matter. Similarly, cerebral microdialysis and cerebral blood flow catheters may have added some useful information about local metabolic changes with alterations in norepinephrine dosing. Finally, even though the response to intravenous PDEI therapy in these two cases was dramatic, it in no way proves superiority or equivalency to standard hypertensive therapy for DCI postaneurysmal SAH. Furthermore, with the absence of cardiac output monitoring during milrinone therapy in these cases, our goal with treatment was to titrate the medication doses to clinical improvement in the neurological examination, as per previously described protocols [15]. It is unknown what the particular hemodynamic changes were during this therapy, which may shed light on more objective targets with milrinone based therapy. Further prospective study with invasive/noninvasive determination of cardiac index during milrinone therapy for DCI is required.

In the setting of DCI post-SAH, hypertensive therapy with norepinephrine may lead to deterioration related to failure of therapy or medication induced vasoconstriction. Overall, we believe that norepinephrine carries the potential for aggravating DCI in certain patients. Prospective studies utilizing genetic profiling, multimodal intracranial monitoring, and neuroimaging need to be conducted in order to determine the effects of various vasopressors on cerebral metabolism and blood flow in post-SAH vasospasm. The role of milrinone in such cases, and for post-SAH vasospasm in general, carries potential and but has yet to be fully determined.

## 4. Conclusions

Hypertensive directed therapy for DCI post-SAH may not lead to symptomatic improvement and potentially may lead to neurological deterioration. Genetic drivers of cerebrovascular response to vasopressors may dictate potential neurological improvement during hypertensive therapy, and further prospective study is warranted utilizing genotyping, multimodal monitoring, and advanced neuroimaging. The role for intravenous vasodilators in post-SAH vasospasm has yet to be determined but is promising.
